# Electrophysiology and arrhythmia care in Romania

**DOI:** 10.1016/j.hroo.2022.08.005

**Published:** 2022-12-16

**Authors:** Andrea-Maria Ursaru, Stefan Bogdan, Fred Kusumoto

**Affiliations:** ∗ Cardiology Department, University Emergency Hospital “Sf. Spiridon”, Iasi, Romania; † “Carol Davila” University of Medicine and Pharmacy, Bucharest, Romania; ‡ Cardiology Department, Elias University Emergency Hospital, Bucharest, Romania; § Department of Cardiovascular Disease, Mayo Clinic, Jacksonville, Florida

**Keywords:** Ablation, Arrhythmia, Cardiac implantable electronic device, Health care system, Romania

## Abstract

Interventional electrophysiological procedures such as catheter ablation and cardiac implantable electronic device (CIED) implantation have become important treatment options for patients with arrhythmia, but delivering state-of-the-art care can be challenging in developing countries. Romania has 19 million people. Throughout Romania, there are 28 centers that can implant CIEDs and 16 centers that can perform catheter ablation, but they are concentrated in the capital of Bucharest. Interventional arrhythmia procedures are performed less frequently than in other European countries (pacemaker: 301/million; implantable cardioverter-defibrillator: 44/million; cardiac resynchronization therapy: 13/million; catheter ablation: 0.8/million). Cardiologists with expertise in arrhythmia care have increased over the past decade and have formed an active organization that has instituted a national registry for interventional arrhythmia procedures and have been active in research and international arrhythmia organizations. Although significant work remains, our electrophysiology community is energized and is working to develop access to the best arrhythmia care for all Romanians.

## Graphical abstract

**Figure:**
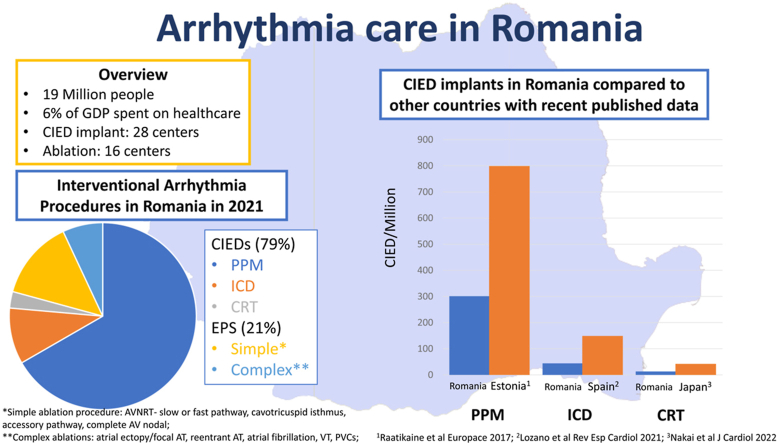

**Key Findings**

▪ Interventional arrhythmia procedures in Romania are performed less frequently than in other European countries, mainly due to financial constraints.
▪ Due to intermittent lack of funds from the National Health Insurance House, cardiac implantable electronic devices (CIEDs) are being reused if they meet the minimum criteria in terms of sterility and electrical performance.
▪ There is a mismatch between the offering and the demand for electrophysiological procedures, leading to very long waiting lists for patients.
▪ Romania has undergone remarkable progress in the field of devices and arrhythmia treatment, with the development of new centers during the last 15 years.
▪ Education of general cardiologists, and devices and arrhythmia specialists is promoted in Romania through activities held regularly. Since 2018, the Romanian Health Ministry has developed specific certifications for electrophysiology and CIED practitioners.

## Introduction

Interventional electrophysiological (EP) procedures such as catheter ablation and cardiac implantable electronic device (CIED) implantation have become important treatment options for patients with arrhythmias that have been incorporated into current worldwide guidelines.^1^, ^2^, ^3^, ^4^ Delivering interventional arrhythmia treatment to the general population can be challenging in developing countries. After the fall of the Communist regime in December 1989, Romania, one of the largest East-European countries, became part of North Atlantic Treaty Organization (NATO) in 2004 and a member of the European Union (EU) in 2007. During the past 15 years, Romania has made tremendous progress toward providing the general population with modern medical treatments that follow current guidelines, including the field of interventional arrhythmia care.

### Current state of arrhythmia care in Romania

According to the Romanian National Institute of Statistics, Romania’s population gradually increased in the latter part of the 20th century, reaching a peak of approximately 23 million in 1990. However, the population has gradually decreased over the past 20 years because of emigration and a net negative birth vs death rate.^5^ The current estimate of the general population based on United Nations data is 19 million, with approximately 12% of the population living in the Bucharest area.^6^

Health care services are provided by 41 districts and the capital (Bucharest) in accordance with centrally defined regulations. Full access to a complete health care package is provided for every tax-paying Romanian citizen via a large network of public hospitals (including university hospitals). Financing is provided by compulsory social health insurance (SHI) (representing approximately 10% of the employee’s gross wage) that the government collects and distributes to the National Health Insurance House (NHIH). The Ministry of Health is responsible for overall governance of the SHI system, and the NHIH is responsible for administering and regulating the National Health Insurance Fund. Despite the compulsory SHI system, an estimated 11% of the general population remains uninsured, mostly in the rural areas. These individuals have access to a limited health care package that covers emergencies, infectious diseases, and care during pregnancy.^7^

**Figure 1:**
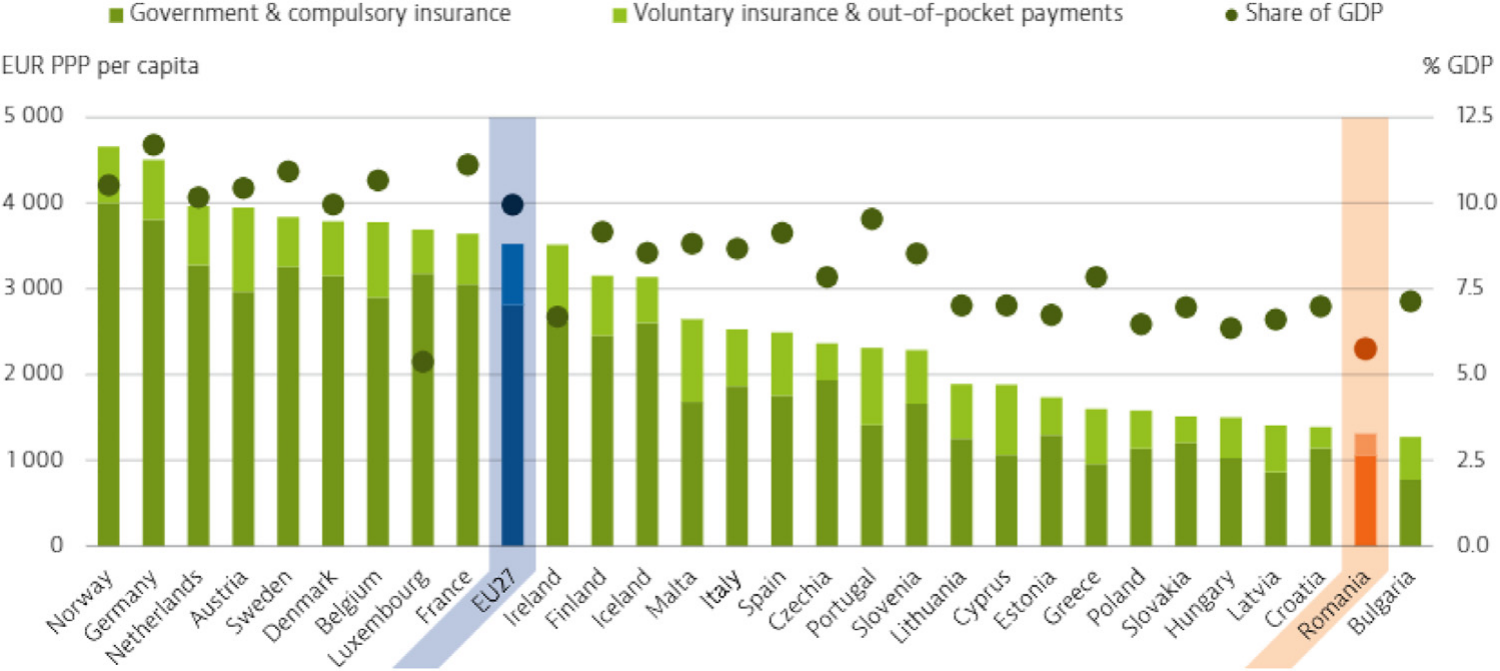
Health spending per capita in Romania is among the lowest among European Union countries. Note: The EU average is weighted. GDP = gross domestic product. (Source: OECD Health Statistics 2021 [data refer to 2019, except for Malta 2018].)

According to a 2019 report, Romania has 532 hospitals (377 public hospitals and 155 private hospitals) and 8.6 million hospital admissions (both inpatient and outpatient), with no specific public data on the impact of arrhythmia care. Romania has significantly increased its health spending since 2015; however, it remains one of the EU countries with the lowest health expenditure, both on a per capita basis and as a proportion of gross domestic product (Figure 1).^7^ Public hospitals—whether primary, secondary, or tertiary; university associated or not—all are reimbursed by the NHIH based on a diagnosis-related group (DRG) system that considers both diagnostic and therapeutic measures. The DRG system, acquired in October 2005 from the Australian government, has been periodically updated but remains outdated in terms of modern arrhythmia treatment, with no specific coding/reimbursement for complex EP procedures or complex CIED implantations.^8^ Private hospitals have increased dramatically over the past 20 years, from 3 in 2000 to 159 in 2020. Private hospitals, which have been estimated to account for 20% of the Romanian national health expenditure, receive most of their reimbursements directly from patients, with the remainder from private medical subscriptions for specific medical services and from the NHIH as described earlier.

**Figure 2:**
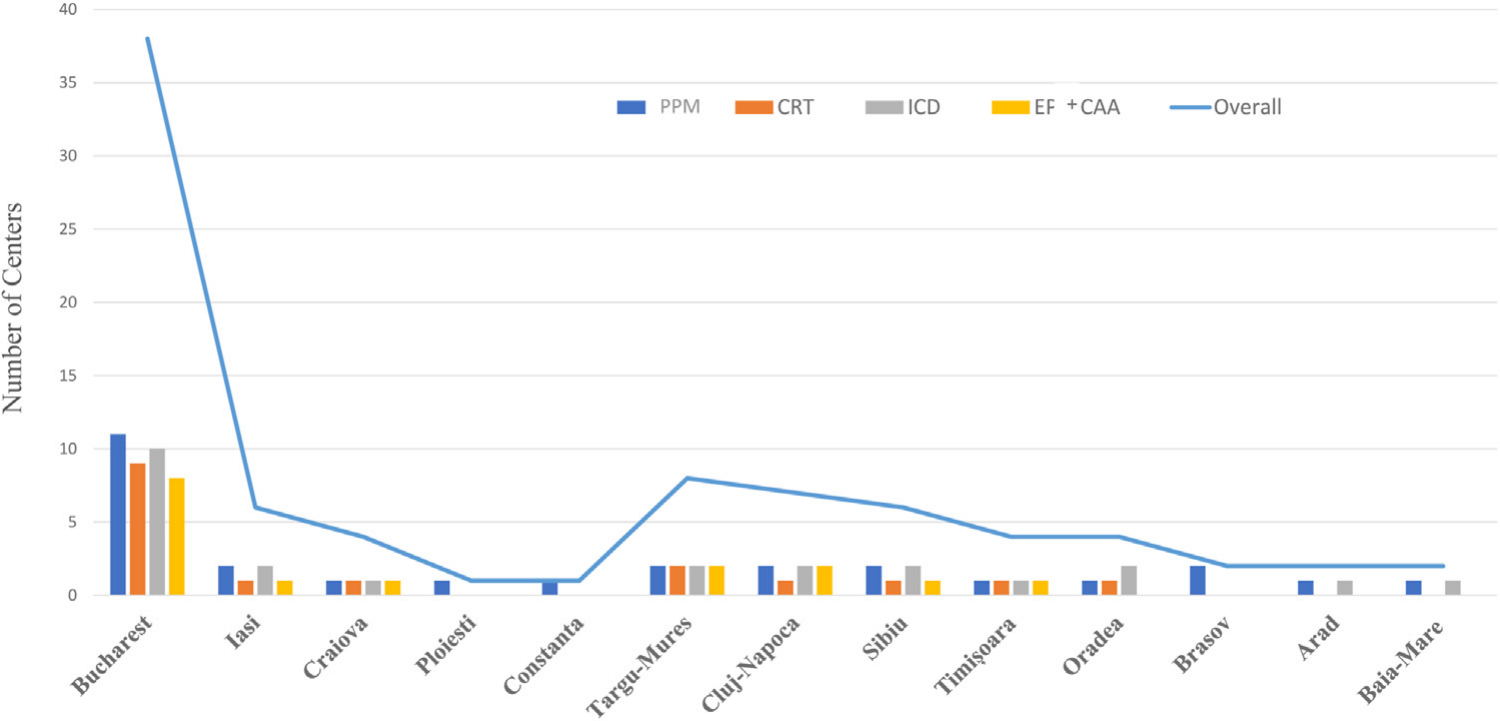
Romanian centers having a valid National Health Insurance House (NHIH) contract for cardiac implantable electronic device (CIED) implantation and electrophysiological procedures in 2022 (by program subtype and region). CAA = complex arrhythmia ablation; CRT = cardiac resynchronization therapy; EP = standard electrophysiological study (with or without radiofrequency ablation); ICD = implantable cardioverter-defibrillator; PPM = permanent pacemaker.
• For non-CIED EP procedures: EP study and complex arrhythmia treatment using ablation therapy (Figure 2 and Supplemental Table 1).

To provide access to complex arrhythmia procedures, tertiary public centers that fulfil the requirements (certified medical staff, infrastructure) must obtain specific funding from the NHIH through a Cardiovascular National Program. During the past 15 years, the network of hospitals accepted by the NHIH for interventional arrhythmia treatment in the setting of the Cardiovascular National Program has increased significantly (almost tripled). Patient access to arrhythmia treatment within the SHI system currently is available at most of the university centers, at larger nonuniversity hospitals, and at a small but increasing number of hospitals in the private sector during the last 2 years. For arrhythmia treatment, there are currently 5 dedicated subprograms within the NHIH Cardiovascular National Program^9^:

• For CIED: Pacemakers (PMs), cardiac resynchronization therapy (CRT), and implantable cardioverter-defibrillators (ICDs) (Figure 2 and Supplemental Table 1)

**Figure 3:**
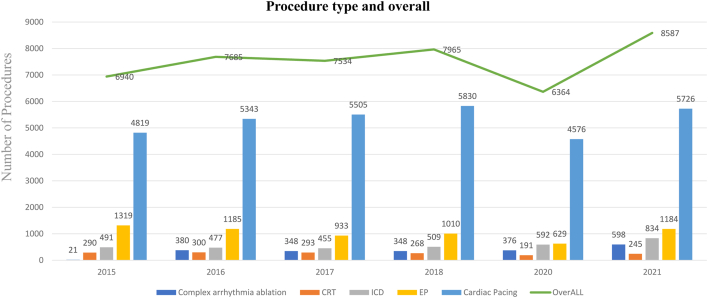
Trends in arrhythmia procedure numbers financed by the NHIH–Cardiovascular National Program between 2016 and 2021 in Romania. ∗Data missing for 2019. Abbreviations as in Figure 2.

NHIH reports have shown that during the year 2020, a total number of 6364 patients benefited from interventional arrhythmia treatment within the NHIH Cardiovascular National Program (Figure 3). Although this number slightly underestimates the real number (probably by 10%–15%) by not considering procedures performed in the private sector without NHIH reimbursement, it is a historical low for the last 7 years.^10^ The main driving factor for the decrease was the coronavirus immunodeficiency disease 2019 (COVID-19) pandemic, during which patient referral and NHIH funding for interventional arrhythmia treatment suffered greatly. In addition, mortality increase during the COVID pandemic was related to undertreatment of comorbidities, including arrhythmias, and active strategies now are required to return to previous levels or to improve arrhythmia care.^11^

### CIEDs: Challenges and opportunities

Europe is characterized by very high regional variations in the rates of PM, ICD, and CRT device implantation.^12^ Romania, which is considered a developing southeastern European country,^13^ has low implantation rates compared with the rest of the continent. The main reason for the low implant rates has been socioeconomic pressures, with patient and physician preferences representing minor factors. Progress during the last decade has been observed, with a significant increase in the number of procedures performed. The average CIED implantation rate rose substantially between 2015 and 2018. After a dramatic drop in procedure rates early during the COVID-19 pandemic, with the lowest figures in the year 2020, hospitals have adapted to new safety protocols, and the rates are now slowly returning to those before the pandemic, with a substantial increase in numbers in 2021 (Figure 3).

Reimbursement for CIEDs in Romania is supported by the NHIH through the 3 sections of the Cardiovascular National Program (Figure 2 and Supplemental Table 1)^14^: for cardiac pacing, NHIH supports PM implant; for CRT, NHIH reimburses only for CRT-P devices; and for ICDs, NHIH also reimburses for CRT-D devices. According to the NHIH, the average cost (in U.S. dollars) for the CIED only (not including associated implant costs) is approximately $670 for PM, $2840 for ICD (median cost for single-/dual-chamber ICD and CRT-D, with no cost differentiation available), and $1850 for CRT-P.^15^ In Romania, patients who receive a CIED are hospitalized with at least an overnight stay because of the lack of reimbursement for outpatient care, which increases the burden of costs. Most CIEDs are reimbursed by public health insurance, and an additional 10% are supported through private subscriptions that have CIED coverage or are purchased directly by the patient. Some private hospitals are also benefiting from NHIH funds, with the addition of hospitalization costs that are supported by the patient. However, the disbursement of NHIH funds is unpredictable; usually the funds are distributed every month, and sometimes quarterly, depending on the availability of financial resources. The distribution among implanting centers corresponds to previous volume, so the endorsement of funds is very low for recently created centers, thus slowing the development of, and becoming a barrier for, new centers. Moreover, the majority of implanting centers are concentrated within university hospitals in big cities in Romania. The majority are located within the capital, leaving vast regions of the country without access to advanced arrhythmia care. For example, the northeastern part of Romania has approximately one-quarter of the country’s population but only 2 of the 28 implanting centers. Fortunately, there has been a considerable increase in the number of implanting centers in the last decade, and patient access to interventional therapy has improved significantly. However, smaller public hospitals continue to have difficulty in accessing health care resources, in many cases limited by the lack of infrastructure necessary for CIED implant.

Several important issues remain. The first is financial constraints and irregularity. Because of the intermittent lack of funds and the high burden of symptomatic bradyarrhythmia, CIEDs are being reused if they meet minimum criteria in terms of sterility and electrical performance. Use of resterilized devices in Romania dates back in the year 2000.^16^ Although a national standardized regulation regarding reuse policy does not yet exist, the National Association for Patients Protection in Romania recognizes and encourages the use of functional resterilized devices, given the lack of complications and adverse events reported by patients who have received implants. Multiple studies performed by 1 center in Romania have proven that reusing CIEDs is feasible and safe, and represents a viable alternative when new devices are not available.^16^, ^17^, ^18^ The humanitarian implications of implanting reused devices are important, considering the inability of available funds to cover the need for new CIEDs, especially high-cost ICDs and CRTs. Although reuse is a viable solution for patients in need of the limited number of CIEDs available in Romania, there is reticence on the part of implanting physicians to use resterilized devices. This reluctance constrains patient access to essential therapy despite published studies proving that reused CIEDs are as safe as new devices with regard to the risk of infection or malfunction rates.^16^, ^17^, ^18^ The possibility of using resterilized CIEDs is further limited by the inability of some implanting centers across the country to perform ethylene oxide sterilization, which is a method widely used to sterilize heat- or moisture-sensitive medical equipment without damaging it during the sterilization process.

The second issue is the lack or inappropriate reimbursement that accounts for the development of important clinical procedures. For example, implantable loop recorders are unavailable or are underused in most centers in Romania despite the utility of these devices in elucidating the etiology of syncope or detecting isolated arrhythmias following ablation procedures. Only a small number of patients receive the devices, although in a cost-to-benefit analysis the health care costs necessary for treatment of the potentially avoidable complications could exceed the cost of an implantable loop recorder.^19^ In addition, subcutaneous ICDs and leadless PMs are not available or are available on a very limited basis. Unfortunately, all device monitoring (direct CIED monitoring or remote monitoring) is not reimbursed by the national health care system.

Finally, the development of a strategy for a centralized registry for cardiovascular health, and specifically for CIED implants, would be extremely helpful as a continuous process for quality improvement. As with any registry, careful implementation to accurately identify demographics, comorbidities, baseline parameters, and outcomes, with consideration of unintended consequences of procedures performed with nonpunitive intent, is essential.

### Ablation: Challenges and opportunities

The biggest challenge today regarding ablation in Romania is making EP procedures more accessible to arrhythmia patients who meet guideline indications. Despite a steady increase in interventional EP activity during the last 20 years (Figure 3), in 2016 Romania reported 0.6 catheter ablation centers per million inhabitants, which recently has improved to 0.8 but remains the lowest in the EU.^20^ Reimbursement by the NHIH Cardiovascular National Program for both simple and complex procedures has helped in recent years, but further efforts are required.

EP procedures are funded through the NHIH Cardiovascular National Program as described for CIEDs. Funding for EP procedures is provided in advance by the NHIH Cardiovascular National Program on a quarterly basis to each center, with the specific amounts based on historical volumes and existing supplies. Centers can apply for supplemental funding, which greatly depends on available NHIH funds. Ablation procedures constitute a very small percentage of total procedures for arrhythmia care (Figure 3). Currently approximately 30 senior electrophysiologists perform ablations in Romania, with a total of 1800 EP procedures performed annually, one-third of which are classified as complex (cryoballoon ablation or ablation using 3-dimensional mapping) (SB, personal estimate).

Given the increased awareness among general cardiologists regarding EP procedures, arrhythmia patients are referred much more quickly for ablation today than they were 10 years ago. The end result is a mismatch between the offering and the demand for EP procedures, leading to very long waiting lists. Although patients with arrhythmias that require urgent treatment (ventricular tachycardia [VT] storm; pre-excited atrial fibrillation; tachycardia-induced cardiomyopathies) are treated preferentially, those with mainly a quality-of-life indication for ablation must wait 6 to 18 months, depending on the center and type of procedure. This situation is not ideal given recent consistent data that have shown the importance of early treatment of atrial fibrillation for reducing adverse clinical outcomes.^20^

Solutions to this problem will require training of future electrophysiologists and dedicated and trained paramedical staff, development of new EP centers, and increased and greater availability of funding for EP procedures.^21^^,^^22^

### Education and professional organizations

In Romania, there are approximately 2500 cardiologists, with fewer than 300 performing interventional procedures. The Romanian Health Ministry has developed a national accreditation system for physicians, and in 2018 specific certifications for EP and cardiac pacing were introduced. A physician can be accredited as a specialist for CIED and/or EP after a minimum of 1 year of training at a national accredited center.^23^ After proving a minimum number of procedures, the physician must pass a written test and a practical evaluation in front of an examination board to become certified for the chosen subspecialty. However, given the small number of high-volume centers and the limited time in training, 1 year usually is not enough to prepare an operator to act independently. Therefore, Romanian physicians interested in complex arrhythmia care commonly go abroad to acquire additional training and time under the supervision of a senior certified practitioner in order to further develop uniform, high-level theoretical knowledge and practical skills. Most of the senior electrophysiologists currently working in Romania have been trained abroad (Spain, Germany, France, Italy, Israel) and now offer state-of-the-art EP interventions for Romanian arrhythmia patients, including VT ablation in the setting of VT storm and atrial fibrillation ablation.

The main arrhythmia-specific professional organization in Romania is the Romanian Working Group of Arrhythmias (RWGA), which is part of the Romanian Society of Cardiology (RSC). The RWGA was founded in September 2002 and currently has 149 members (including fellows).^24^ The RWGA is an active participant in activities sponsored by the European Heart Rhythm Association (EHRA). The majority of arrhythmia health care practitioners in Romania are members of EHRA and have contributed to EHRA clinical guidelines and the EHRA Atrial Fibrillation Registries.^25^^,^^26^ In 2021, the RWGA created a national registry of arrhythmia-related procedures in Romania. Currently, participation is voluntary, and the data are not publicly available. At this time, data based directly on hospital reimbursements of procedures provide more complete information, but it is hoped that future health care policy decisions will increase the completeness and accuracy of the registry, which will provide greater details and nuance.

Education of general cardiologists, and device and arrhythmia specialists is promoted in Romania through activities held regularly by the National Conference of Working Groups and at the annual National Congress of Cardiology, but also at dedicated conferences focused on arrhythmias including Actualitati in Aritmii Cardiace–Novelties in Cardiac Arrhythmias (ARCA), the International Iasi Arrhythmia Forum (IAF), and the Complex Arrhythmia Ablation Workshop.

Continued education in the field of arrhythmia care is essential for all physicians in Romania, not only for electrophysiologists and CIED implanting physicians but also for all cardiologists regardless of subspecialty, because arrhythmia management for many patients is critical, as is overcoming the reluctance of physicians to refer patients for procedures considered elective (eg, ICD in the primary prevention of sudden cardiac death). Although barriers to implementation of the guidelines in clinical practice are mainly financial, continued education remains critical, and learning opportunities are necessary to promote future development of arrhythmia interventional therapy and to provide the best treatment to patients.

## Conclusion

Romania has undergone remarkable progress in the field of arrhythmia treatment, from mainly conservative strategies in the mid-1990s to complex CIED implantations and arrhythmia ablation today. The development of interventional arrhythmia centers during the last 15 years offers fair coverage for most of the population, but new centers as well as more procedures performed in currently active centers are still required. To that purpose, the training of new electrophysiologists and dedicated paramedical staff is crucial, as is improvement of funding/reimbursement for arrhythmia procedures. Close collaboration with regulatory institutions, patient education, and political lobbying all are important for reaching an important common goal: offering our arrhythmia patients the best medical treatment according to guidelines in a reasonable time frame.

## Funding Sources

This research did not receive any specific grant from funding agencies in the public, commercial, or not-for-profit sectors.

## Disclosures

The authors have no conflicts to disclose.

## Authorship

All authors attest they meet the current ICMJE criteria for authorship.
