# Supplementary material for: Electrophysiology and arrhythmia care in Romania
Source: Heart Rhythm O2. 2022 Dec 16;3(6Part B):793–8. doi: 10.1016/j.hroo.2022.08.005 (PMC9795282; doi:10.1016/j.hroo.2022.08.005)
Supplement: Supplementary Table 1 [file mmc1.docx]

**Tables**

**Table 1.** List of Romanian Centers having a valid contract in 2022 with the National Health Insurance House for CIED implantation and EP procedures, by program subtype and region.

|  | **PPM** | **CRT**  (only CRT-P devices) | **ICD**  (includes CRT-D devices) | **EPS+CAA** |
| --- | --- | --- | --- | --- |
| ***Total number of centers*** | 28 | 17 | 24 | 16 |
| ***Bucharest area*** | 11 | 9 | 10 | 8 |
| -          Institutul de Urgenta pentru Boli Cardiovasculare « Prof. Dr. C.C. Iliescu » | X | x | X | x |
| -          Spitalul Universitar de Urgenta | X | x | X | x |
| -          Spitalul Clinic de Urgenta | X | x | X | x |
| -          Spitalul Universitar de Urgenta Militar Central « Dr. Carol Davila » | X | x | X | x |
| -          Spitalul Clinic de Urgenta “Sf. Ioan” | X |  |  |  |
| -          Spitalul Clinic Colentina | X | x | X | x |
| -          Spitalul Clinic de Urgenta “Bagdasar-Arseni” | X |  |  |  |
| -          Spitalul Universitar de Urgenta Elias | X | x | X | x* |
| -          Spitalul de Urgenta “Prof. Dr. Agrippa Ionescu” | X | x | X |  |
| -          Spitalul Sanador | X | x | X | x |
| -          Centrul Medical Policlinico di Monza | X | x | X | x |
|  |  |  |  |  |
| ***Wallachia (Southern part of Romania)*** | 3 | 1 | 1 | 1 |
| -          Spitalul Clinic Judetean de Urgenta Craiova | X | x | X | x |
| -          Spitalul Judetean de Urgenta Ploiesti | X |  |  |  |
| -          Spitalul Clinic Judetean de Urgenta Constanta | X |  |  |  |
|  |  |  |  |  |
| ***Transylvania (North-western part of Romania)*** | 12 | 6 | 11 | 6 |
| -          Spitalul Clinic Judetean de Urgenta Targu Mures | X | x | X | x |
| -          Institutul de Urgenta pentru Boli Cardiovasculare si Transplant Targu Mures | X | x | X | x |
| -          Institutul de Boli Cardiovasculare Timisoara | X | x | X | x |
| -          Institutul Inimii de Urgenta pentru Boli Cardiovasculare « Nicolae Stancioiu » Cluj-Napoca | X | x | X | x |
| -          Spitalul Clinic de Recuperare Cluj-Napoca | X |  | X | x |
| -          Spitalul Judetean de Urgenta Baia Mare | X |  | X |  |
| -          Spitalul Clinic Judetean de Urgenta Sibiu | X |  | X | x |
| -          Spitalul Clinic Judetean de Urgenta Arad | X |  | X |  |
| -          Spitalul Clinic Judetean de Urgenta Brasov | X |  |  |  |
| -          Spitalul Clinic Judetean de Urgenta Oradea | X |  | X |  |
| -          Clinicile ICCO Brasov | X |  |  |  |
| -          Clinica Polisano Sibiu | X | x | X |  |
| -          Spitalul Pelican Oradea |  | x | X |  |
|  |  |  |  |  |
| ***Moldova (North-eastern part of Romania)*** | 2 | 1 | 2 | 1 |
| -          Institutul de Boli Cardiovasculare “Prof. Dr. George I.M. Georgescu” Iasi | X | x | X | x |
| -          Spitalul Clinic Judetean de Urgenta “Sf. Spiridon” Iasi | X |  | X |  |

CAA – complex arrhythmia ablation; CIED – cardiac implantable electronic device; CRT – cardiac resynchronization therapy; EP – interventional electrophysiology; EPS – standard electrophysiological study and radiofrequency ablation; ICD – implantable cardioverter defibrillator; PPM – permanent pacemaker;

* Recently accepted by NHIH – will receive funding starting July 1^st^ 2022
